# Esophageal stenosis after chemotherapy for breast cancer: A case report

**DOI:** 10.1097/MD.0000000000029045

**Published:** 2022-03-18

**Authors:** Zhen-Fei Ou, Lin-Lin Ren, Xiao-Yan Yin, Cui-Ping Zhang, Yong-Hong Xu, Cong-Cong Min

**Affiliations:** ^a^ *Department of General Medicine, the Affiliated Hospital of Qingdao University, Qingdao, China,* ^b^ *Department of Gastroenterology, the Affiliated Hospital of Qingdao University, Qingdao, China.*

**Keywords:** breast cancer, chemotherapy, esophageal stenosis

## Abstract

**Rationale:**

Esophageal stenosis after chemotherapy in breast cancer patients is rare. Distinguishing esophageal stenosis from esophageal metastasis caused by breast cancer is important.

**Patient concerns and diagnosis:**

A 62-year-old woman diagnosed with advanced breast cancer and no distant metastases gradually developed skin changes, oral ulcers and mucosal injures after four cycles of chemotherapy. Dysphagia was the most severe symptom that greatly affected the patient’s quality of life. Ultimately, esophageal stenosis and ulceration were confirmed by serial radiological examinations and endoscopic biopsy.

**Interventions:**

Due to difficulties in eating orally, the patient was initially placed on a nasogastric tube in order to improve her nutritional status. Simultaneously, she was administered powerful proton pump inhibitors. She underwent modified radical mastectomy for breast cancer after her nutritional status improved. However, the patient was still suffering from severe dysphagia after more than 4 months of follow-up. Subsequently, she underwent removable esophageal stent implantation after after unsuccessful attempts to dilate her esophagus.

**Outcomes:**

The dysphagia symptoms were immediately alleviated to a certain degree, and the dilated cavity of the upper esophagus showed slight retraction.

**Lessons:**

Esophageal stenosis is very infrequent in patients with breast cancer after chemotherapy. It needs to be. distinguished from esophageal metastasis caused by breast cancer. Esophageal stent implantation may provide benefits in terms of both symptom control and survival in patients with severe esophageal structures.

## 1. Introduction

Patients with advanced tumors often undergo neoadjuvant chemotherapy to shrink the tumors and alleviate metastatic cells in early stages to facilitate subsequent treatments, such as surgery and radiotherapy. Paclitaxel, cyclophosphamide, and doxorubicin hydrochloride liposome injections are intravenous chemotherapy drugs that are commonly used in patients with breast cancer. These drugs have been reported to cause changes in the skin and mucosa; however, their effects on the esophageal mucosa have rarely been reported.^[1]^ Herein, we report an uncommon case of chemotherapy-induced esophageal stricture.

## 2. Case report

A 62-year-old woman presented with dysphagia and retrosternal pain that had persisted for 2 months. The patient was diagnosed with right breast cancer (T2N1M0) 4 months previously. The patient did not undergo surgery for metastatic breast cancer in the right axillary lymph nodes. Therefore, she was administered TEC regimen chemotherapy including albumin paclitaxel (400mg), doxorubicin hydrochloride liposome injection (50 mg), and cyclophosphamide (0.8 g) by intravenous drip at an interval of 21 days for four cycles. She developed severe mouth ulcers, painful swallowing and large areas of skin pigmentation with overlying scaliness after each chemotherapy session (Fig. 1A, B). Over time, she experienced progressively worsening dysphagia and vomiting after eating. Ultimately, she could swallow only liquid food. Owing to the above symptoms and malnutrition, chemotherapy was discontinued. (The drug manufacturer of albumin paclitaxel and doxorubicin hydrochloride liposome injection was CSPC Pharmaceutical Group Limited. The manufacturer of cyclophosphamide was purchased from Baxter Oncology GmbH. The specifications of albumin paclitaxel, doxorubicin hydrochloride liposome injection, and cyclophosphamide were 100mg, 20mg, 0.2 g each.)

**Figure:**
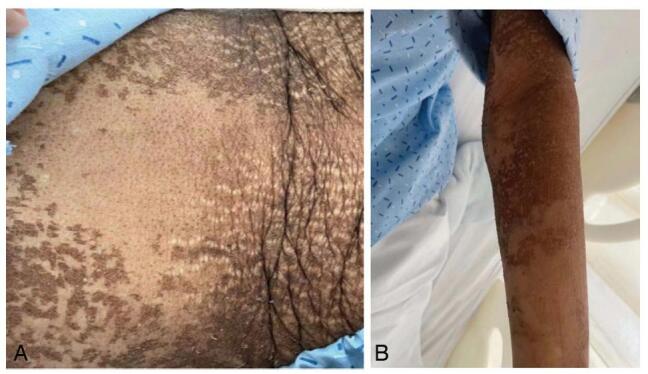
**Figure 1.** Skin changes Large areas of black pigmentation on the whole body with overlying scaliness, accompanied by chapped skin. (A) Abdomen (B) Upper left limb.

Physical examination revealed malnutrition and hair loss after chemotherapy. Surprisingly, her skin throughout her body showed large areas of skin pigmentation with overlying scaliness and skin cracks. Laboratory results revealed mild hypoproteinemia and hypokalemia. Her carcinoembryonic antigen level was 6.02ng/mL (normal range, 0-3.4). Upper gastrointestinal radiography showed significant stenosis of the middle and lower third of the esophagus with a filling defect and stiffening of the canal wall, suggestive of esophageal cancer (Fig. 2A). Computed tomography of the chest showed a narrow esophageal cavity in the middle and lower esophagus (Fig. 2B) and dilation of the upper esophagus (Fig. 2C). Upper gastrointestinal endoscopy revealed that the lumen of the lower esophagus (35 cm from the incisor) was severely narrow with ulceration above the stenosis (Fig. 3A, B). Histological results revealed inflammatory fibrinoulcerative and necrotizing esophagitis with granulation tissue formation and no obvious tumor cells (Fig. 4A, B).

**Figure:**
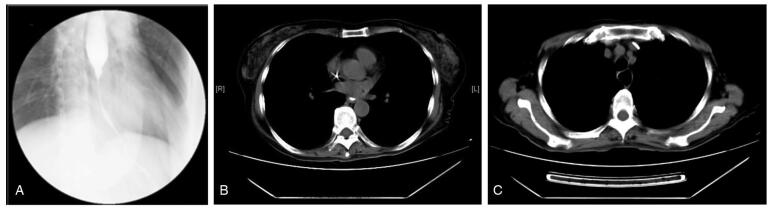
**Figure 2.** Upper gastrointestinal radiography and computed tomography showed esophageal stenosis. A filling defect in the middle and lower esophagus, stiffening of the wall, and narrowing of the lumen (A). Esophageal stricture was seen in the middle and lower esophagus (B). The cavity of the upper esophagus was dilated. (C).

**Figure:**
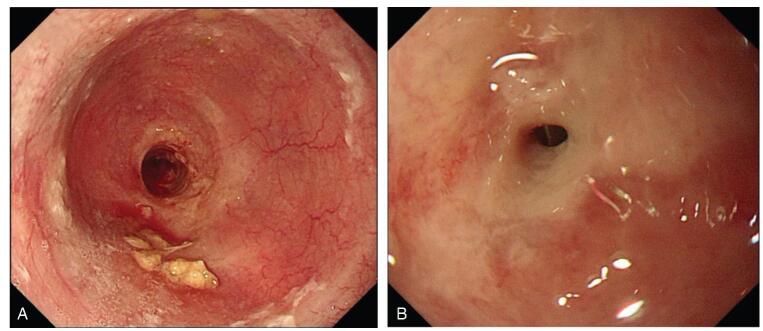
**Figure 3.** Upper gastrointestinal endoscopy performances. Esophageal stenosis was observed 35 cm from the incisor with dilation of the upper esophageal cavity (A). Ulcerative changes of esophageal mucosa above the stenosis (B).

**Figure:**
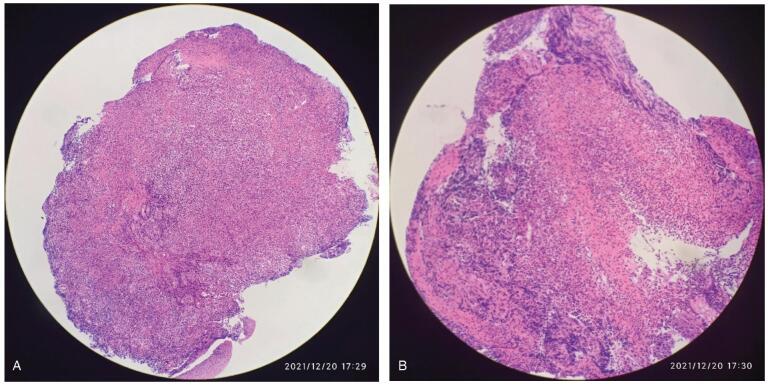
**Figure 4.** Histological features. Inflammatory fibrinoulcerative and necrotising esophagitis with granulation tissue formation with no obvious tumor cells (A: H&E 100×; B: H&E 200×).

According to the skin damage and oral ulcers after each chemotherapy session, as well as endoscopic manifestations and pathological features, the esophageal stenosis was considered to be caused by esophageal mucosal injury due to chemotherapy drugs.

Considering the greater risk of esophageal perforation and tracheoesophageal fistula caused by esophageal dilatation, a nasogastric tube was inserted to start enteral nutrition support. Simultaneously, she was administered proton pump inhibitor. The nasogastric tube was unobstructed, and the patient had no nausea or vomiting. The patient’s right breast tumor slightly shrank after chemotherapy and was considered stable. Subsequently, she underwent a modified radical mastectomy for right breast cancer under general anesthesia. She was still suffering from severe dysphagia after more than 4 months of follow-up. Upper gastrointestinal radiography showed stenosis of the middle and lower thirds of the esophagus did not improve significantly. Subsequently, the patient underwent removable esophageal stent implantation (Fig. 5A) after esophageal dilation failure. Her dysphagia symptoms were immediately alleviated to a certain degree, and the dilated cavity of the upper esophagus showed slight retraction (Fig. 5B).

**Figure:**
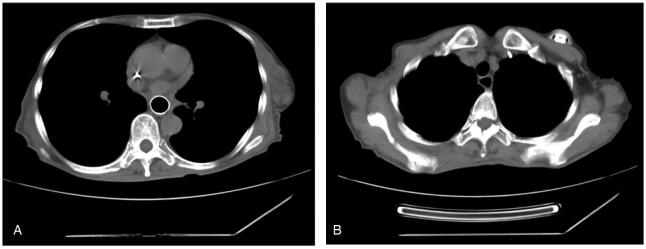
**Figure 5.** Computed tomography after esophageal stent placement. An esophageal stent (red arrow) was implanted in the lower esophageal stricture (A). Improvement of dilation of the upper esophagus after stent placement (B).

## 3. Discussion

Breast cancer is currently the most common cancer among women in China, and in the European Union, half of cancer patients are diagnosed with breast cancer each year.^[2]^ We first suspected esophageal stenosis caused by metastasis from breast cancer, as metastatic foci within the lymph nodes were found in the patient. Esophageal metastasis of primary breast cancer is rare, with nearly all the literature on this topic consisting of case reports.^[3]^ A case was reported in a patient who was successfully cured of breast cancer after chemotherapy and mastectomy, and developed metastatic breast cancer within the esophagus 19 years later.^[4]^ Esophageal metastases are rare in breast cancer patients, most of whom are between 50 and 70 years of age. The diseasefree interval is usually 5-10 years, with most metastases occurring in the mid-thoracic esophagus.^[5]^ Esophageal metastasis from breast cancer most often involves the middle or lower third of the esophagus.^[3]^ Esophageal metastasis always originates from the outside inward, and metastasis to the mucosal layers is extremely rare. Therefore, previous studies have reported endoscopic findings of an esophagus with a normal mucosa.^[3]^ Endoscopic ultrasound-guided fine-needle aspiration can improve the biopsy-positive rate. In our case, the esophageal stricture occurred in the middle or lower third of the esophagus. However, the patient had an obvious esophageal mucosal injury manifesting as ulcers and local luminal stenosis. Histopathological examination showed no malignant tumor cells, but chronic ulcerative lesions with granulation tissue and inflammatory infiltrates. In addition, worsened dysphagia occurred soon after chemotherapy for breast cancer, which differed from previous reports.

Doxorubicin, cyclophosphamide, and paclitaxel (Taxol) are currently the main treatment options for new adjuvant therapies for breast cancer.^[5]^ The mechanism of action of Taxol, which is mainly derived from the yew tree *Picea abies*, involves inhibition of mitosis. The main skin and mucosal reactions include alopecia, mucositis, and erythema, which usually occur after drug discontinuation.^[1]^ Matsuura et al reported a case of severe esophageal stricture after radiotherapy for thoracic metastases following bevacizumabpaclitaxel combination chemotherapy in a patient with recurrent breast cancer.^[6]^ Cyclophosphamide is a nitrogen mustard drug that plays an important role in breast cancer and blood disorders.^[7]^ Jensen et al reported erythema (n = 10, 22%) and ulceration (n = 7, 16%) in 45 breast cancer patients treated with cyclophosphamide and epirubicin, respectively.^[8]^ Doxorubicin is a commonly used anthracycline antitumor drug in clinical practice. The main mechanism of action is the inhibition of DNA synthesis. Akhtar et al showed that fluorouracil, doxorubicin, and cyclophosphamide treatment caused more vomiting and mucositis than paclitaxel and carboplatin treatment.^[9]^ Our patient developed lesions on the skin, mucosa, and oral ulcers after each chemotherapy session. Her chest computed tomography scan before chemotherapy showed that the esophageal wall was normal, with no esophageal stenosis. We consider that these chemotherapy drugs may also have an effect on the esophageal mucosa and that dysphagia is usually unnoticeable until this effect becomes more severe. However, esophageal mucosal ulcerations and strictures caused by chemotherapy have not been previously reported. Although the three chemotherapeutic drugs that cause these damages cannot be determined, as they all can cause oral ulcers and mucosal damage, there may be a synergistic effect with the combination of the three drugs. First, we placed a nasal feeding tube to improve patient nutrition. Subsequently, she underwent modified radical mastectomy for right breast cancer under general anesthesia. Her dysphagia did not improve significantly after four months; therefore, she underwent removable esophageal stent implantation. Subsequently, the patient’s dysphagia symptoms were partially relieved, and she was discharged from the hospital.

## 4. Conclusion

Esophageal stenosis after chemotherapy in patients with breast cancer is rare and less commonly reported. Previous reports have revealed esophageal metastasis from breast cancer usually after 5 to 10 years of disease-free interval. In our case, the esophageal stricture was considered to have been caused by chemotherapy. Skin and mucosal injuries can occur shortly after chemotherapy in breast cancer. In severe cases, it can even cause esophageal stenosis. Therefore, physicians should pay close attention to the digestive symptoms at the time of chemotherapy and promptly manage these symptoms. The patient needs to be followed-up with regular gastroscopy and endoscopic ultrasonography.

## Author contributions

**Funding acquisition:** Cong-Cong Min.

**Investigation:** Lin-Lin Ren, Xiao-Yan Yin.

**Methodology:** Yong-Hong Xu.

**Project administration:** Cui-Ping Zhang.

**Supervision:** Cui-Ping Zhang, Xiao-Yan Yin.

**Writing** - **original draft:** Zhen-Fei Ou.

**Writing** - **review & editing:** Zhen-Fei Ou, Cong-Cong Min.
